# Correction: A value-based model of job performance

**DOI:** 10.1371/journal.pone.0319222

**Published:** 2025-02-07

**Authors:** The PLOS ONE Staff

In Table 2, the values in the column ‘δ‘ are incorrect. Please see the correct Table 2.

**Table 2:** Probability to deviate from norms.

Types	Deviation from Norms	δ
C	low	1/3
O	**high**	1
SE	medium	2/3
ST	medium	2/3

The publisher apologizes for the error.
