# Supplementary material for: A value-based model of job performance
Source: PLoS One. 2022 Jan 27;17(1):e0262430. doi: 10.1371/journal.pone.0262430 (PMC8794576; doi:10.1371/journal.pone.0262430)
Supplement: S1 File — (PDF) [file pone.0262430.s001.pdf]

Supporting Information

S1 Additional tables

Table S1.1. Agent parameters.

Types	δ	γ	ϕ		ρ	
			Trusting	Controlling	Individual	Group
C	$1/3$	0	$0.5s_t^*\delta$	$-0.5s_t^*\delta$	0	0
O	1	0	$-0.5s_t^*\delta$	$0.5s_t^*\delta$	0	0
SE	$2/3$	$-0.5c_t^*\delta$	0	0	$-0.5c_t^*\delta$	$0.1c_t^*\delta$
ST	$2/3$	$0.5c_t^*\delta$	0	0	$-0.1c_t^*\delta$	$0.5c_t^*\delta$

Table S1.2. Model parameters.

Parameter	Description	Value
N	number of agents in the model	100
steps	simulation period	3000
numpeers	number of random peers (local envs)	8
dist	probability distribution of agent types	$(0.25, 0.25, 0.25, 0.25)$
κ	degree of task interdependence	0.5
τ	time budget	10
w	hourly wage	1
h	rate of adjustment to social norms	0.1
Σ	trusting vs. neutral vs. controlling stance	$\{0, 0.5, 1\}$
μ	fixed wage vs. PFP plans	$\{0, 1\}$
λ	individual vs. collective PFP plans	$\{0, 1\}$

S2 Environment similarity

Two additional environments for social norms have been tested, both having a local scope:

1. *Neighbours*: norms are local and agents look only at what their closest peers do. We assume a Moore neighbourhood of range 1, such that each agent is surrounded

by a constant set of peers denoted by M, with $M \subseteq N$:

$$c^*_{i,t} = \frac{\sum_{j \epsilon M} h\ c_{j,t-1} - c^*_{i,t-1}}{M} \tag{1}$$

2. *Random*: norms are local and agents look at what other n random agents from the whole population do ($n \subseteq N$):

$$c^*_{i,t} = \frac{\sum_{j \epsilon n} h\ c_{j,t-1} - c^*_{i,t-1}}{n} \tag{2}$$

The end results of the simulations are consistent throughout all the theorised norm environments, suggesting that the two limited scopes of social norms ($Neighbours$ and *Random*) converge towards the trend of the *Global* scope. Indeed, the dynamics of output are exactly the same over the three environments in the long-run. All data and plots for the $Neighbours$ and *Random* scope of social norms can be (re-)created and compared via the provided and openly accessible code.

The similarity between the three chosen environments *Global*, $Neighbours$ and *Random* can be seen in table S2.3. Since the scope of social norms is outside of the influential sphere of the management, the displayed similarity between the three variants might be interpreted in a positive way: When using aggregate output (i.e. sum of the mean output of all agents over all steps) as a reference point, the scope of social norms has very little impact across all nine scenarios.

Scenario	Env	Aggregate Output (Y)	Mean ($\bar{Y}$)	$\%(Y-\bar{Y})$	Std	$\%Y$
Base	Global	10040.794	10141.231	−0.01	320.443	0.032
	Neighbours	10499.861	10141.231	0.035	320.443	0.031
	Random	9883.038	10141.231	−0.025	320.443	0.032
Trusting	Global	13611.428	13530.77	0.006	456.053	0.034
	Neighbours	13941.112	13530.77	0.03	456.053	0.033
	Random	13039.769	13530.77	−0.036	456.053	0.035
Controlling	Global	1340.591	1368.129	−0.02	44.353	0.033
	Neighbours	1419.293	1368.129	0.037	44.353	0.031
	Random	1344.503	1368.129	−0.017	44.353	0.033
Cooperative	Global	10870.74	10944.831	−0.007	83.216	0.008
	Neighbours	11034.865	10944.831	0.008	83.216	0.008
	Random	10928.889	10944.831	−0.001	83.216	0.008
Competitive	Global	2475.983	2603.259	−0.049	194.091	0.078
	Neighbours	2826.652	2603.259	0.086	194.091	0.069
	Random	2507.141	2603.259	−0.037	194.091	0.077
Trustcoop	Global	11062.765	11136.027	−0.007	81.096	0.007
	Neighbours	11223.166	11136.027	0.008	81.096	0.007
	Random	11122.151	11136.027	−0.001	81.096	0.007
Trustcomp	Global	2930.3	3096.448	−0.054	257.988	0.088
	Neighbours	3393.657	3096.448	0.096	257.988	0.076
	Random	2965.387	3096.448	−0.042	257.988	0.087
Contrcoop	Global	10247.299	9941.719	0.031	683.108	0.067
	Neighbours	10418.693	9941.719	0.048	683.108	0.066
	Random	9159.166	9941.719	−0.079	683.108	0.075
Contrcomp	Global	920.012	934.558	−0.016	25.985	0.028
	Neighbours	964.558	934.558	0.032	25.985	0.027
	Random	919.103	934.558	−0.017	25.985	0.028

Table S2.3. Similarity between environments.

S3 Sensitivity analysis

All sensitivity analyses have been conducted with the base setting of 50 replicates (each equalling to a 3000 step simulation) per each of the nine scenarios, leading to a total number of 20250 simulation runs. The results are condensed across the replicates by computing mean results for each collected variable, leading to 405 datasets, one for each unique parameter constellation.

Three main parameter sweep tests have been performed.

Test 1: *dist*

First, we tested for the effects of different probability distributions of agent types. To address this issue, we go beyond the uniform distribution of agents of the baseline scenario to check for agents' time allocation decisions after increasing the relative share of one value group to 70% and lowering the share of each other group to 10%. Table S3.4 summarises our hypotheses for each possible type distribution.

Higher share	Hypotheses
C-distribution	*H1.1.1* A negative correlation between a trusting corporate culture and the production times of C agents;<br>*H1.1.2* A positive correlation between a trusting corporate culture and the shirking times of C agents;<br>*H1.1.3* Less production and more shirking time across every value group ∀ trusting scenarios;<br>*H1.1.4* More production and less shirking time across every value group ∀ controlling scenarios.
O-distribution	*H1.2.1* A positive correlation between a trusting corporate culture and the production times of O agents;<br>*H1.2.2* A negative correlation between a trusting corporate culture and the shirking times of O agents;<br>*H1.2.3* More production and less shirking time across every value group ∀ trusting scenarios;<br>*H1.2.4* Less production and more shirking time across every value group ∀ controlling scenarios.
SE-distribution	*H1.3.1* A positive correlation between a cooperative corporate culture and the production times of SE agents;<br>*H1.3.2* A negative correlation between a cooperative corporate culture and the cooperation times of SE agents;<br>*H1.3.3* Less production and more shirking time across every value group ∀ cooperative scenarios;<br>*H1.3.4* More production and less cooperation time across every value group ∀ competitive scenarios.
ST-distribution	*H1.4.1* A negative correlation between a cooperative corporate culture and the production times of ST agents;<br>*H1.4.2* A positive correlation between a cooperative corporate culture and the cooperation times of ST agents;<br>*H1.4.3* Less production and more cooperation time across every value group ∀ cooperative scenarios;<br>*H1.4.4* Less production and more shirking time across every value group ∀ competitive scenarios.

Table S3.4. Distribution probability and hypotheses.

Tables S3.5-S3.8 show that some hypotheses at table S3.4 cannot be confirmed as a result of the role that the time constraint plays in agents' behavioural decisions. The

following paragraphs provide brief explanations about why some propositions do not hold per each probability distribution of agent types.

Hypothesis *H1.1.3* is confirmed with the exception of O-agents in a *Trustcoop* scenario: O-agents tend to produce more relative to the Uniform distribution case (compare numbers in bold at table S3.5). In a C-distribution, the high share of conservative employees boosts both the shirking and the cooperative norm, as a consequence of the negative correlation between individual tasks and trusting scenarios satisfied at *H1.1.1*. However, a trusting and cooperative management reduces O-agents' shirking and, in the context of already high cooperation, leaves O-agents with more time to devote to individual tasks.

Higher Share	Scenario	C-type			O-type			SE-type			ST-type		
		Prod	Shirk	Coop	Prod	Shirk	Coop	Prod	Shirk	Coop	Prod	Shirk	Coop
	Base	3.8077	3.107	3.0853	3.8176	3.1015	3.0809	4.1492	3.1072	2.7436	3.4656	3.1066	3.4279
	Trusting	6.22	0.4265	3.3535	6.308	0.3371	3.3549	6.6136	0.4042	2.9822	5.8697	0.4042	3.7261
	Controlling	2.9654	6.6314	0.4032	2.0184	7.6115	0.3702	2.6969	6.9491	0.354	2.6142	6.9451	0.4406
	Cooperative	2.481	0.3434	7.1755	2.8259	0.3275	6.8466	3.1855	0.3395	6.475	1.4176	0.3187	8.2637
Uniform	Competitive	6.2932	3.373	0.3338	6.2934	3.373	0.3336	6.3669	3.3733	0.2598	6.2639	3.3727	0.3634
	Trustcoop	2.5229	0.2372	7.24	**2.9089**	0.1815	6.9096	3.2438	0.223	6.5332	1.4517	0.2125	8.3358
	Trustcomp	9.2382	0.4272	0.3346	9.3278	0.3378	0.3345	9.3347	0.4049	0.2605	9.2309	0.4048	0.3642
	Contrcoop	2.3816	0.8148	6.8036	2.5801	0.9419	6.478	3.0099	0.8509	6.1392	1.348	0.7991	7.8529
	Contrcomp	3.0584	6.7167	0.2248	2.0795	7.7077	0.2127	2.7883	7.0379	0.1738	2.7211	7.036	0.2428
	Base	3.6081	3.2044	3.1876	3.6222	3.1972	3.1805	3.9633	3.203	2.8336	3.2564	3.2038	3.5398
	Trusting	1.2164	8.2546	0.5291	3.3558	6.1343	0.5099	1.9556	7.5846	0.4597	1.8533	7.5772	0.5695
	Controlling	6.1951	0.4727	3.3322	6.083	0.5833	3.3337	6.5373	0.5004	2.9623	5.7988	0.5004	3.7008
	Cooperative	2.4286	0.8513	6.7201	2.7495	0.8111	6.4394	3.09	0.8414	6.0686	1.4214	0.7898	7.7888
C-distribution	Competitive	5.8314	3.3535	0.8151	5.83	3.3549	0.815	6.0135	3.3522	0.6343	5.7595	3.3533	0.8872
	Trustcoop	1.1553	7.971	0.8737	**3.2301**	5.9315	0.8384	1.8908	7.3327	0.7764	1.6866	7.298	1.0154
	Trustcomp	1.2739	8.3418	0.3843	3.4288	6.1981	0.3731	2.0426	7.6634	0.294	1.931	7.6598	0.4092
	Contrcoop	2.7048	0.3709	6.9243	2.9196	0.4466	6.6338	3.3556	0.3909	6.2535	1.5981	0.3802	8.0217
	Contrcomp	8.7106	0.4735	0.8159	8.5996	0.5845	0.8159	8.8639	0.5012	0.6349	8.6108	0.5012	0.888

Table S3.5. Average production, shirking and cooperation time by C-distribution.

Hypotheses *H1.2.1.* and *H1.2.3* are not satisfied since all agents appear to produce less in a *Trustcomp* scenario. In such a scenario, two opposite forces are in place: (i) shirking should decrease as driven by O-agents, and (ii) competitive rewards should discourage cooperative tasks. These two forces should allow agents to have more time for individual tasks. But this appears not to be the case because (i) time devoted to individual tasks is already very high (around 90% of the available time), and (ii) lower shirking allows for higher cooperation due to the stochastic effect of residual available

time.

Proposition *H1.2.4.* must be partially rejected. Indeed, ST-agents produce more in a *Contrcoop* scenario. These agents experience a low cooperative attitude because shirking activities are predominant due to the high share of O-agents. Since ST-agents are the only ones positively responding to cooperative bonuses, and since they are a minority, they are not able to boost cooperation and to counteract the influence of O-types on the behaviour of the population.

Higher Share	Scenario	C-type			O-type			SE-type			ST-type		
		Prod	Shirk	Coop	Prod	Shirk	Coop	Prod	Shirk	Coop	Prod	Shirk	Coop
Uniform	Base	3.8077	3.107	3.0853	3.8176	3.1015	3.0809	4.1492	3.1072	2.7436	3.4656	3.1066	3.4279
	Trusting	6.22	0.4265	3.3535	6.308	0.3371	3.3549	6.6136	0.4042	2.9822	5.8697	0.4042	3.7261
	Controlling	2.9654	6.6314	0.4032	2.0184	7.6115	0.3702	2.6969	6.9491	0.354	2.6142	6.9451	0.4406
	Cooperative	2.481	0.3434	7.1755	2.8259	0.3275	6.8466	3.1855	0.3395	6.475	1.4176	0.3187	8.2637
	Competitive	6.2932	3.373	0.3338	6.2934	3.373	0.3336	6.3669	3.3733	0.2598	6.2639	3.3727	0.3634
	Trustcoop	2.5229	0.2372	7.24	2.9089	0.1815	6.9096	3.2438	0.223	6.5332	1.4517	0.2125	8.3358
	Trustcomp	**9.2382**	0.4272	0.3346	**9.3278**	0.3378	0.3345	**9.3347**	0.4049	0.2605	**9.2309**	0.4048	0.3642
	Contrcoop	2.3816	0.8148	6.8036	2.5801	0.9419	6.478	3.0099	0.8509	6.1392	**1.348**	0.7991	7.8529
	Contrcomp	3.0584	6.7167	0.2248	2.0795	7.7077	0.2127	2.7883	7.0379	0.1738	2.7211	7.036	0.2428
O-distribution	Base	4.0852	2.9724	2.9424	4.0899	2.9695	2.9406	4.4127	2.971	2.6163	3.7597	2.9718	3.2684
	Trusting	6.5361	0.1084	3.3555	6.5574	0.0857	3.3569	6.9136	0.1027	2.9837	6.1698	0.1028	3.7274
	Controlling	2.2591	7.6195	0.1214	1.6493	8.2438	0.1068	2.0839	7.8115	0.1047	2.0584	7.8118	0.1298
	Cooperative	3.3152	0.7423	5.9426	3.4154	0.7283	5.8564	3.8469	0.7412	5.4119	2.0768	0.7291	7.1941
	Competitive	5.8732	3.327	0.7998	5.8739	3.3266	0.7995	6.0523	3.3253	0.6224	5.8031	3.3264	0.8705
	Trustcoop	3.6945	0.1077	6.1978	3.8074	0.0851	6.1074	4.2535	0.1021	5.6444	2.3966	0.1021	7.5013
	Trustcomp	**9.076**	0.1084	0.8156	**9.0986**	0.0858	0.8156	**9.2626**	0.1027	0.6347	**9.0094**	0.1028	0.8877
	Contrcoop	2.2541	7.6133	0.1326	1.6465	8.2374	0.1161	2.078	7.8051	0.1169	**2.0419**	7.8036	0.1545
	Contrcomp	2.2639	7.6245	0.1116	1.6521	8.2491	0.0988	2.0984	7.817	0.0846	2.0653	7.8173	0.1174

Table S3.6. Average production, shirking and cooperation time by O-distribution.

Hypothesis *H1.3.3.* must be rejected because all agents produce more in all cooperative scenarios, with the exception of O-types in a *Contrcoop* setting. In the SE-distribution, both norms are driven by SE agents, being the majority. In all cooperative scenarios, SE agents tend to be less cooperative. This means that they could spend more residual time both on shirking and on personal activities. However, SE-types do not directly react through means of shirking, hence they will delegate more time to individual tasks. Their behaviour directly impacts C-agents since they have the lowest probability to deviate from social norms. ST agents tend to cooperate less with respect to the Uniform distribution since the cooperative norm is lower, hence they devote more time to personal activities. O-agents tend to shirk more and cooperate less, having the highest probability

to deviate from social norms, leaving more time to individual activities. The same is not true for O-agents in a *Contrcoop* scenario: They devote more than 72% of their time to shirking activities and, as a consequence, they do not have enough time to perform individual tasks.

Moreover, all agents appear to produce less in a *Trustcomp* scenario, contrary to what we expected in *H1.3.4*. Since competitive rewards strongly undermine cooperation, especially in the context of a SE-distribution, and since individual tasks are residual, a reduction in cooperation is immediately accompanied by an increase in shirking activities. This leaves agents with a lower amount of time available for personal tasks. The increase in shirking derives from a statistical effect, i.e. from the downward pressure SE agents exerts on the cooperation norm, and is not driven by other agents' reaction to the management style.

Higher Share	Scenario	C-type			O-type			SE-type			ST-type		
		Prod	Shirk	Coop	Prod	Shirk	Coop	Prod	Shirk	Coop	Prod	Shirk	Coop
Uniform	Base	3.8077	3.107	3.0853	3.8176	3.1015	3.0809	4.1492	3.1072	2.7436	3.4656	3.1066	3.4279
	Trusting	6.22	0.4265	3.3535	6.308	0.3371	3.3549	6.6136	0.4042	2.9822	5.8697	0.4042	3.7261
	Controlling	2.9654	6.6314	0.4032	2.0184	7.6115	0.3702	2.6969	6.9491	0.354	2.6142	6.9451	0.4406
	Cooperative	**2.481**	0.3434	7.1755	**2.8259**	0.3275	6.8466	**3.1855**	0.3395	6.475	**1.4176**	0.3187	8.2637
	Competitive	6.2932	3.373	0.3338	6.2934	3.373	0.3336	6.3669	3.3733	0.2598	6.2639	3.3727	0.3634
	Trustcoop	**2.5229**	0.2372	7.24	**2.9089**	0.1815	6.9096	**3.2438**	0.223	6.5332	**1.4517**	0.2125	8.3358
	Trustcomp	**9.2382**	0.4272	0.3346	**9.3278**	0.3378	0.3345	**9.3347**	0.4049	0.2605	**9.2309**	0.4048	0.3642
	Contrcoop	**2.3816**	0.8148	6.8036	2.5801	0.9419	6.478	**3.0099**	0.8509	6.1392	**1.348**	0.7991	7.8529
	Contrcomp	3.0584	6.7167	0.2248	2.0795	7.7077	0.2127	2.7883	7.0379	0.1738	2.7211	7.036	0.2428
SE-distribution	Base	6.4601	3.3702	0.1696	6.4615	3.369	0.1695	6.4792	3.3699	0.1509	6.442	3.3696	0.1884
	Trusting	8.8051	1.0252	0.1697	9.0212	0.8092	0.1696	8.8777	0.9714	0.1509	8.8405	0.9711	0.1884
	Controlling	3.6414	6.191	0.1676	2.5486	7.2849	0.1665	3.3281	6.5229	0.149	3.2916	6.5224	0.186
	Cooperative	**6.3524**	3.3681	0.2795	**6.354**	3.3667	0.2793	**6.3775**	3.3677	0.2548	**6.291**	3.3674	0.3413
	Competitive	6.5497	3.3717	0.0786	6.5509	3.3705	0.0786	6.5672	3.3714	0.0614	6.5434	3.3711	0.0855
	Trustcoop	**8.6954**	1.0249	0.2797	**8.9115**	0.809	0.2795	**8.774**	0.9711	0.2549	**8.6877**	0.9708	0.3415
	Trustcomp	**8.8959**	1.0255	0.0786	**9.1119**	0.8095	0.0786	**8.967**	0.9716	0.0614	**8.9431**	0.9713	0.0855
	Contrcoop	**3.5519**	6.1831	0.265	2.464	7.275	0.261	**3.2441**	6.5147	0.2412	**3.1628**	6.5141	0.3232
	Contrcomp	3.7283	6.1934	0.0783	2.6341	7.2879	0.078	3.4135	6.5254	0.0611	3.39	6.5249	0.0851

Table S3.7. Average production, shirking and cooperation time by SE-distribution.

Contrary to what was hypothesised in *H1.4.4.*, all agent-types shirk less in all competitive scenarios. The low level of shirking for all agents is driven by the higher tendency to cooperate of the majority of the population due to the prevalence of ST-agents. About 80% of their time is devoted to cooperative activities, compared to roughly 3.6% in the Uniform distribution case. This greatly boosts the cooperative norm and

reduces the amount of time available for shirking activities. Therefore, when the share of ST agents is high, competitive rewards seem to have two positive effects: they (i) do not undermine a generally high cooperative norm, and (ii) reduce shirking behaviour.

Higher Share	Scenario	C-type			O-type			SE-type			ST-type		
		Prod	Shirk	Coop	Prod	Shirk	Coop	Prod	Shirk	Coop	Prod	Shirk	Coop
	Base	3.8077	3.107	3.0853	3.8176	3.1015	3.0809	4.1492	3.1072	2.7436	3.4656	3.1066	3.4279
	Trusting	6.22	0.4265	3.3535	6.308	0.3371	3.3549	6.6136	0.4042	2.9822	5.8697	0.4042	3.7261
	Controlling	2.9654	6.6314	0.4032	2.0184	7.6115	0.3702	2.6969	6.9491	0.354	2.6142	6.9451	0.4406
	Cooperative	2.481	0.3434	7.1755	2.8259	0.3275	6.8466	3.1855	0.3395	6.475	1.4176	0.3187	8.2637
Uniform	Competitive	6.2932	**3.373**	0.3338	6.2934	**3.373**	0.3336	6.3669	**3.3733**	0.2598	6.2639	**3.3727**	0.3634
	Trustcoop	2.5229	0.2372	7.24	2.9089	0.1815	6.9096	3.2438	0.223	6.5332	1.4517	0.2125	8.3358
	Trustcomp	9.2382	**0.4272**	0.3346	9.3278	**0.3378**	0.3345	9.3347	**0.4049**	0.2605	9.2309	**0.4048**	0.3642
	Contrcoop	2.3816	0.8148	6.8036	2.5801	0.9419	6.478	3.0099	0.8509	6.1392	1.348	0.7991	7.8529
	Contrcomp	3.0584	**6.7167**	0.2248	2.0795	**7.7077**	0.2127	2.7883	**7.0379**	0.1738	2.7211	**7.036**	0.2428
	Base	1.6475	0.1857	8.1668	2.3345	0.1729	7.4926	2.7385	0.1817	7.0798	1.3323	0.1735	8.4942
	Trusting	1.645	0.1773	8.1778	2.3634	0.1316	7.505	2.746	0.1647	7.0893	1.3369	0.1576	8.5054
	Controlling	1.6522	0.1959	8.1519	2.3034	0.2221	7.4745	2.7304	0.2027	7.0669	1.3278	0.1931	8.4791
	Cooperative	1.2816	0.0955	8.6229	2.1267	0.0883	7.7851	2.3031	0.0931	7.6039	0.8492	0.083	9.0677
ST-distribution	Competitive	2.0091	**0.2925**	7.6984	2.5401	**0.2751**	7.1847	3.8129	**0.2889**	5.8982	1.6577	**0.28**	8.0623
	Trustcoop	1.2788	0.0957	8.6256	2.1401	0.0706	7.7893	2.3051	0.0885	7.6064	0.8502	0.0792	9.0706
	Trustcomp	2.0112	**0.2636**	7.7252	2.5902	**0.1976**	7.2122	3.8344	**0.247**	5.9186	1.6699	**0.2401**	8.09
	Contrcoop	1.2851	0.0952	8.6197	2.1133	0.1071	7.7796	2.3011	0.098	7.6009	0.8483	0.0872	9.0645
	Contrcomp	2.011	**0.3343**	7.6546	2.4789	**0.3832**	7.1379	3.7858	**0.3493**	5.8648	1.6448	**0.3379**	8.0173

Table S3.8. Average production, shirking and cooperation time by ST-distribution.

Test 2: h

The second parameter sweep test is performed on the norms influence parameter h. We take into account three possible values of the parameter (0.1, 0.5 and 1.0) with 0.1 as the reference point. We test for the impact of different h with the following hypothesis:

H2.1 There is a negative correlation between h and the variance of the deviation from the cooperation and shirking norms. A higher (lower) h leads to a lower (higher) variance deviation from norms across all employee distributions.

Fig S3.1 shows that the variance of the deviations from both social norms, cooperation and shirking, decreases more drastically with higher values of h.

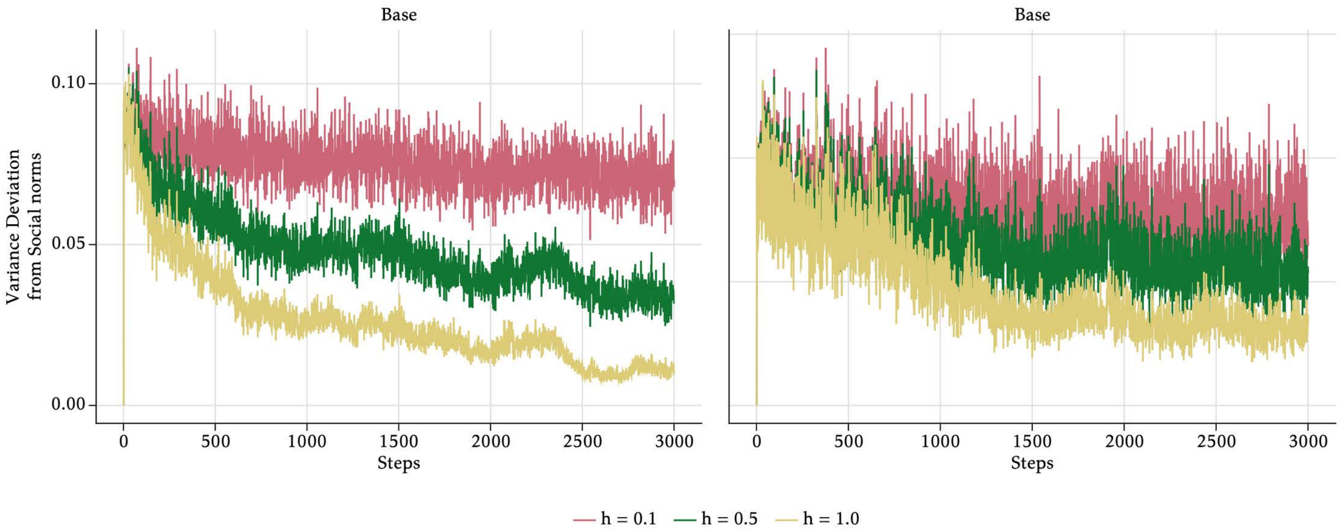

Fig S3.1 Impact of h on the variance of the deviations from social norms.

In line with *H2.1*, the decreasing variance occurs over the long-run. This effect is due to the faster integration of more recent behaviour into the social norm.

From step 250 onwards, the variance deviation from both social norms becomes more divergent among the three possible values of h. Strong changes in the norms around that simulation period might have left O-agents, those with the highest probability to deviate, with less room for deviations. This in turn pushes down the variance for all values of h, with the same happening sooner the higher the influence of norms on behaviour.

Test 3: κ

Last, the degree of task interdependence has been analysed for values of κ equal to 0, 0.5 (baseline scenario) and its maximum amount 1. In this regard, we formulate the following hypotheses:

H3.1 A high (low) κ leads to an overall decrease (increase) in output.

H3.2 A high (low) κ leads to a proportionally weaker decrease in output of ST (SE) agents in comparison to the other three value groups.

To test the two hypotheses above, we computed the average difference in output with respect to the two extreme values of κ. The overall average output under a low-κ regime is 32% greater than the output obtained with higher values of κ, independently of the agent-types and of the strategies implemented by the management. This suggests that *H3.1* cannot be rejected: A higher degree of task interdependence is worst performing in terms of corporate output when agents and scenarios are treated in aggregate terms. *H3.2* cannot be rejected because the average output of ST (SE) agents decreases by 18% (43%) between the extreme κ regimes. C and O agents experience an intermediate drop in output, respectively by 35% and 32%, as κ increases. However, when looking at the impact of changing κ per agent-types and scenarios, table S3.9 shows the existence of a positive (negative) correlation between average output and κ under a *Cooperative* (*Competitive*) management style. This is to be expected given the higher partial contribution of cooperative activities to output triggered by an increase in κ (see equation 1). When the management style is either *Trusting* or *Controlling*, higher values of κ lead to decreasing average output, further confirming the general expectations of *H3.1*.

In scenarios including reward schemes, the overall picture is not as straightforward. Reward schemes have a very strong effect on the overall output in relation to κ, both in cases with or without monitoring. While *Trusting* and *Cooperative* scenarios generally

show decreasing (increasing) output trends with higher κ, it might be expected that a combination of those measures would counter each other out. However, *Trustcoop* exhibits an even stronger increase in output compared to a solely *Cooperative* scenario. Inverse observations can be made for the relations between *Trusting*, *Competitive* and the combined *Trustcomp* scenario. The same pattern emerges when a *Controlling* management style is combined with the reward schemes. These results strengthen our conclusion about the positive (negative) correlation between cooperative (competitive) incentives and output, discussed in Section 3.5: The higher the degree of task interdependence, the wider the divergence between the two financial bonuses.

Scenario	κ	C-type			O-type			SE-type			ST-type		
		Mean	Std	Median	Mean	Std	Median	Mean	Std	Median	Mean	Std	Median
	0.0	3.8077	0.207	3.8436	3.8176	0.3109	3.8313	4.1492	0.2491	4.1692	3.4656	0.2634	3.4888
Base	0.5	3.402	0.0529	3.4066	3.2829	0.1446	3.2902	3.5208	0.0872	3.5244	3.1906	0.1043	3.1969
	1.0	3.0844	0.0914	3.0816	3.0845	0.0914	3.0816	3.0879	0.0915	3.085	3.0809	0.0913	3.0781
	0.0	6.22	0.7391	6.5348	6.308	0.6117	6.4908	6.6136	0.7127	6.8768	5.8697	0.7082	6.1273
Trusting	0.5	4.5369	0.3021	4.6668	4.542	0.2587	4.6213	4.6737	0.2859	4.7799	4.3887	0.3036	4.5008
	1.0	3.3542	0.0413	3.3486	3.3542	0.0413	3.3485	3.3579	0.0414	3.3523	3.3504	0.0413	3.3448
	0.0	2.9654	0.2069	2.9882	2.0184	0.3581	2.0066	2.6969	0.2921	2.6849	2.6142	0.3168	2.6318
Controlling	0.5	0.5559	0.8697	0.0911	0.3665	0.6191	0.0598	0.5152	0.8421	0.0812	0.4681	0.7313	0.0812
	1.0	0.3919	0.8772	0.0028	0.3922	0.8777	0.0028	0.3924	0.8782	0.0028	0.3915	0.8762	0.0028
	0.0	2.481	0.1991	2.4901	2.8259	0.3791	2.8251	3.1855	0.3168	3.2001	1.4176	0.2878	1.3982
Cooperative	0.5	4.1111	0.3087	4.193	3.7477	0.4338	3.7761	4.3854	0.4178	4.4561	2.2337	0.3396	2.2281
	1.0	7.1904	0.7984	7.4697	7.1937	0.7992	7.4734	7.1974	0.7993	7.4771	7.1794	0.7979	7.4586
	0.0	6.2932	0.6561	6.5539	6.2934	0.6825	6.5032	6.3669	0.5239	6.5357	6.2639	0.7248	6.5207
Competitive	0.5	0.8662	0.9231	0.3795	0.8566	0.9069	0.3779	0.8842	0.9629	0.379	0.8538	0.8985	0.3782
	1.0	0.3225	0.6435	0.0219	0.3225	0.6435	0.0219	0.3233	0.645	0.0219	0.3222	0.6429	0.0219
	0.0	2.5229	0.1758	2.5128	2.9089	0.3879	2.8939	3.2438	0.281	3.2436	1.4517	0.3085	1.4237
Trustcoop	0.5	4.1646	0.2241	4.2052	3.8311	0.3618	3.8281	4.4499	0.3345	4.4784	2.2792	0.3417	2.2704
	1.0	7.2548	0.7295	7.4734	7.2581	0.7303	7.4772	7.2619	0.7303	7.4809	7.2437	0.7291	7.4623
	0.0	9.2382	1.4224	9.9222	9.3278	1.2661	9.9337	9.3347	1.2368	9.9301	9.2309	1.4428	9.9228
Trustcomp	0.5	1.0097	1.0226	0.4659	1.0272	1.0572	0.466	1.0334	1.0702	0.4666	1.0055	1.0141	0.4657
	1.0	0.3233	0.6449	0.022	0.3233	0.6449	0.0219	0.3241	0.6464	0.022	0.323	0.6443	0.0219
	0.0	2.3816	0.2778	2.433	2.5801	0.5663	2.6704	3.0099	0.4586	3.0958	1.348	0.2952	1.33
Contrcoop	0.5	3.9159	0.5033	4.1317	3.4363	0.735	3.6325	4.1371	0.6529	4.3457	2.1168	0.3876	2.1204
	1.0	6.8186	1.1225	7.4499	6.8219	1.1231	7.4536	6.8253	1.1237	7.4575	6.808	1.1215	7.4389
	0.0	3.0584	0.192	3.0401	2.0795	0.3763	2.0604	2.7883	0.3845	2.7361	2.7211	0.2759	2.7164
Contrcomp	0.5	0.3915	0.749	0.0782	0.2719	0.5726	0.0498	0.3749	0.7603	0.0692	0.3468	0.6763	0.069
	1.0	0.2134	0.606	0.0021	0.2136	0.6061	0.0021	0.214	0.6074	0.0021	0.2133	0.6054	0.0021

Table S3.9. Statistics for output variable by κ.
