# Supplementary material for: A value-based model of job performance
Source: PLoS One. 2022 Jan 27;17(1):e0262430. doi: 10.1371/journal.pone.0262430 (PMC8794576; doi:10.1371/journal.pone.0262430)
Supplement: Attachment — Submitted filename: Response to Reviewers.pdf [file pone.0262430.s002.pdf]

Response to Reviewers
"A value-based model of job performance"

(PONE-D-21-29609)

We would like to sincerely thank the referees for their comments and suggestions.
Their critical assessment of our work has been precise and insightful and has really contributed to the improvement of our manuscript.
We address their concerns in the following pages. The comments of the *Reviewer 2* are discussed on pages 9-11.

Reviewer 1

Comments

Reviewer Point P 1.1 — The main improvements I have listed for the manuscript are regarding the clarity with which information is delivered. In addition the manuscript is quite lengthy. I received 30 pages to review, which I guess after better figure placement would be reduced to 25. Perhaps it is worth considering grouping information more efficiently (e.g., I've noticed that the aspects of heterogeneity are repeated several times, or I've noticed that there are lengthy justifications in the methodology section which could be made shorter, especially given their mentioning in the introduction)

Reply: We thank the referee for the improvements proposed. Rereading the paper after some time, we found some points indeed a bit unclear. We hence followed your suggestion to provide clearer math wherever possible, see P 1.9. Moreover, since we deviate a lot from standard assumptions in economics, we consider it necessary to provide sufficient verbal explanations, which makes it hard to consistently shorten the paper (P 1.6). The journals requirements unfortunately do not allow us to delegate some explanations to footnotes. We did our best to shorten the paper wherever possible, especially Sections 2.2 and 2.3.

Reviewer Point P 1.2 — I applaud the authors for making the source code of their work available openly online, which is the only way one can ensure true reproducibility of scientific work. I believe the authors should mention this in Sect. 4, as this makes their model immediatelly accessible to others for further research. Something like "Our model is available as open source code[cite], and could be integrated in decision making protocols based on agent heterogeneity in corporate settings."

Reply: Thank you very much for your enthusiastic support.
We completely agree about the importance of public availability of source codes. Not only does this practice ensure the possibility to reproduce already existing scientific researches, also implicitly enhancing their quality, but

it also serves the purpose of building a concrete bridge between theoretical knowledge and application.
Following your suggestion, we included the following sentence in Section 4:

> "Our model has been made available as open source code (here) to encourage reproducibility and open collaboration, both necessary to deepen our understanding of heterogeneous agents' decision making processes in corporate settings."

Reviewer Point P 1.3 — The introduction of the paper is well written and gives a good overview of what has been done and the standing view points on the subject. What I found it lacking is that it does not make it crystal clear of what are the specific research questions to be answered by this paper. At 1/3rd of the introduction, the authors point out the novelty of their approach "agents differ in personal values". So the questions are somewhat implicitly assumed as "how does this new approach affect the socioeconomic dynamics of the model". But one should be clearer and explicit in the introduction about the research questions, especially given that the remaining 2/3rds of the introduction continue to review existing literature, but don't come back at what the authors actually want to do.

Reply: Thank you for raising this point.
At page 2 we state the following:

> "This paper presents a theoretical agent-based model that treats monetary incentives, motivational factors and endogenous social norms as joint determinants of employees' work effort and the resulting company performance. We use the model to answer the question of how incentives set by different remuneration systems affect shirking and cooperative behaviour in different organisational cultures. In particular, we compare the effects of a uniform payment scheme with identical salaries for all employees, an individual reward scheme with personal performance-related incentives and a collective reward scheme, in which incentives depend on group performance. A company's management can partly influence the organisational culture, e.g. by monitoring employees, but culture also evolves endogenously by employees' perception of what constitutes normal behaviour. The paper fills a gap in the research literature, since the influence of corporate culture and remuneration are usually treated in separate strands of the literature."

Moreover, our research question is paraphrased at the beginning of Section 3:

> "Our main research question is how incentives set by different remuneration systems affect shirking and cooperation and hence also output in different organisational culture".

We have adjusted the formatting of the relevant paragraph in the introduction to emphasise its importance, and we added the following sentence at page 3:

> "We use our model to answer the research question: What is the impact of organisational culture and remuneration schemes on corporate performance when employees are heterogeneous in their personal values?"

We hope this solution is acceptable.

Reviewer Point P 1.4 — Given that one of the premises of the paper is that agents are individuals with different values, I was surprised to read in the start of Sect. 2.1 that agent skills and productivity are all identical. I guess this is a simplified assumption the authors made to reduce the number of parameters of

the model. This is okay. One thing I'm wondering is, would the results of the simulations change if the agents did not have identical skills, but rather different skill and productivity levels randomly distributed in an agent population? Can the authors provide some intuitive justification on this, without necessarily re-making lengthy simulations?

Reply: This is a very interesting question which we have also discussed internally during the research process. This simplifying assumption had to be made because our present model does not represent concrete production or knowledge acquisition processes. The abstract nature of our theoretical model does not permit sensible modelling of individual skills or task efficiency, hence these employee characteristics are assumed to be homogeneous. The referee's suggestion to implement a stochastic process would require us to make assumptions about the distribution properties. This would lead to the introduction of stochastic noise as well as additional cognitive load for the readers which arguably does not contribute to the understanding of our fundamental research question. However, it may be of interest to the referee that we are currently developing a more complex version of our underlying model which will include heterogeneous and endogenous productivity levels among other factors.

To be clear about this issue, we changed the sentence at the beginning of Section 2.1 as follows:

> "There are N employees in the company. For simplicity, they are all identical in terms of their productivity and skills, given the abstract nature of the production process we aim at describing, but differ in terms of their values as discussed in the next subsection."

Reviewer Point P 1.5 — Is the agent coupling all-to-all when computing the average t_{jc}? Please make it clear in the paper that there is no spatial structure in this agent model.

Reply: The average variable t_{jc} (which has been changed to $c_{j,t}$, see P 1.14) plays two roles in our model:

1. t_{jc} contributes to individual output $O_{i,t}$, equation 1. In this case, agents are coupled all-to-all, independently of the scope of the social norms;
2. t_{jc} enters the definition of the descriptive social norms, see equations 3 and 4 at page 7. How agents are coupled depends on the scope of the social norms. In the main simulations of the model, we assume that social norms have a *Global* scope. In this case, agents are again coupled all-to-all since t_{jc} is averaged across the whole population (N). In Supporting Information S2, we also test for two local norms environments where t_{jc} is instead averaged over a subset of the population. However, a local norms environment is supposed to impact the computation of the social norm only, not exerting any impact on the t_{jc} calculus of the output function at point 1.

In other words, agents are coupled all-to-all in terms of individual output determination, independently of the norm environment, but they do not experience a *complete interaction network* when forming social norms in the two additional local environments.

About the spatial component, agents are positioned on a $10x10$ grid which does not play a role in our main simulations where we have the case of $Global$ norms. Indeed, the space only becomes relevant in the context of a $Neighbours$ environment, where agents form their social norms on the basis of what n neighbouring agents do. The spatial grid can therefore be understood as a helper structure which enables the easy construction of dense networks (e.g. work teams in a company). To clarify this issue, we added the following sentence at page 16:

> "In all scenarios, the company has a fixed workforce of 100 employees positioned on a $10x10$ grid. However, the spatial component plays a role only when a local environment for social norms is activated, see Supporting Information S2."

Reviewer Point P 1.6 — I am skeptical of the opening paragraph of Sect. 2.2 and also whether it is necessary at all. First of all, Ref. 19, which the entire paragraph is based on, is not a scientific reference published by a scientific publisher, but rather it is a public book. Secondly, it seems that all of this discussion on system1/system2 is not necessary. What is necessary for Sect. 2.2 is the "descriptive norms" assumption, which is already stated in the introduction. In fact, nothing in my perception and understanding of Sect. 2.2 changes once I remove its first paragraph. The only point that system1 is referred to is to justify the inclusion of the stochastic term δ, which, I think, is an inclusion that needs no real justification. If the first paragraph is indeed important, the authors need to do a better job connecting it with the remaining of the section, and also connecting it with more solid scientific references.

Reply: Thank you for this comment.
We believe that the paragraph about System-1/System-2 thinking is necessary, especially in the context of the economic decision making process we aim at describing. When addressing an economic audience, it is not as straightforward as it should be to think about non-rational behaviour. Hence, we do not find ourselves comfortable with deleting that paragraph, as it serves the purpose of justifying why we assume that employees base their decision making on social norms instead of rationally maximising an utility function.
If requested by the reviewer, we are willing to substitute the book for other papers by Kahnemann, even if we consider citing the book as the more elegant approach. Not only has Kahnemann received the Nobel price in economics, but also the book has received 36088 citations on Google Scholar and has been reviewed by many scientific journals.

Reviewer Point P 1.7 — The statistical sampling of the stochastic component of the model is not explained adequately. This comment ties with my technical comment on the mathematical notation of the paper being hard to navigate. The distribution function f_x is presented, but it is not stated clearly and explicitly which of all the presented variables are actually sampled from f_x. Is it Δs_i and Δc_i? The δ are presented as the stochastic components of the model, and hence one would assume that these are sampled from a distribution. But that cannot be true, because later we read "In this baseline case, employees would most likely spend their time according to the norm since the expected deviation is zero." which makes me think that f_x in fact samples entire time intervals instead of Δ.

Reply: Thank you for raising this concern. We completely agree about the inadequate explanations behind the statistical sampling of the model. For this reason, we added the following clarifications in Section 2.2. at page 8:

> "To model the stochastic deviations from the norms we use a triangular distribution (T), which has the convenient property of being bounded between two parameters, a and b. The actual time employees spend on a generic activity at a given time x_t is derived as a random draw from the triangular distribution, that is $x_t \sim T(a, b, m)$, see Figure 1."

We sincerely wish this paragraph clarifies that the probability density function samples entire time intervals. However, we found it convenient not to include anymore the formula defining the generic triangular distribution $f(x)$ in order to include more specific equations directly linked to the behaviour of the postulated economy, see P 1.9.

Reviewer Point P 1.8 — I think the manuscript can benefit from a figure/sketch that has some kind of arrows or basic shapes that map and illustrate visually the main aspects and main formulas of the model. E.g. one mapping would be "heterogeneous properties" to "actual model parameters and functions changed to reflect this heterogeneity", another box would contain the responsiveness to rewards, etc. For example, one box would be "cooperativeness" which would map to "distribution function for values of Δt_c" (or t_c once the authors clarify their notation of what the distribution functions actually sample).

Reply: Following the referee's advice, we included a figure at page 5, at the beginning of Section 2, to facilitate the readers' understanding of the main elements of the model, their relations and their math specifications. For the sake of clarity, we enclose this new figure below.

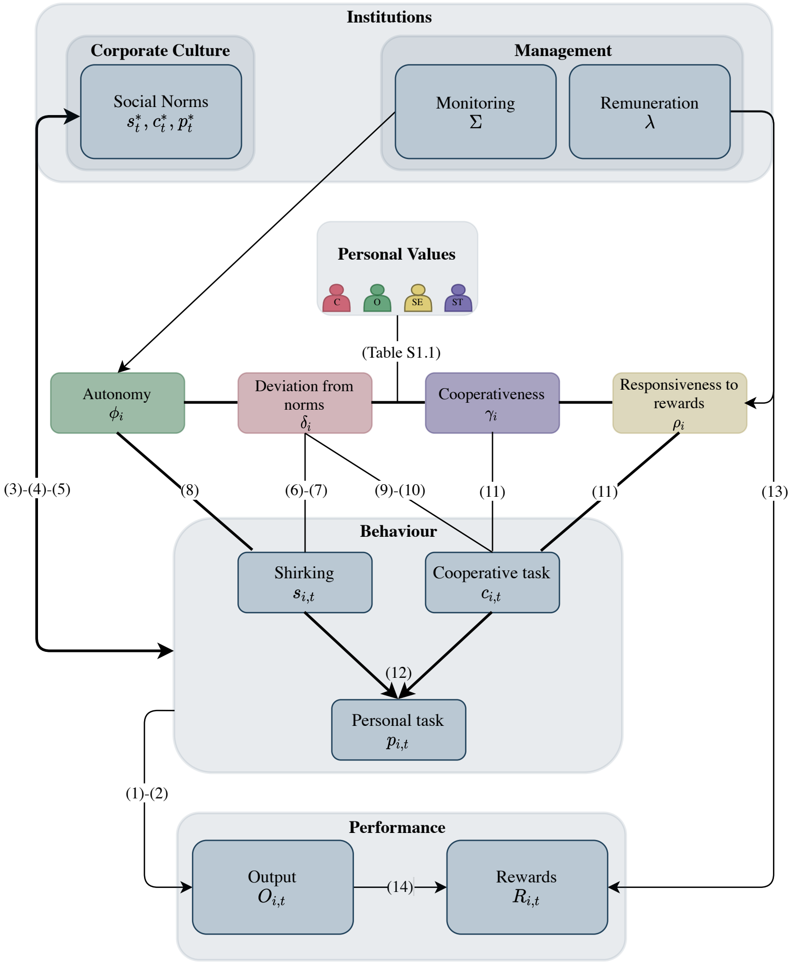

Figure 1: Model overview. The numbers in parenthesis are equation numbers.

We sincerely hope this sketch to be in line with your suggestion.

Reviewer Point P 1.9 — Throughout section 2.3 the authors introduce incrementally parameters (such as γ, ϕ, ...) that alter the main probability distribution function f_x that models all stochastic aspects of the model. However, these parameters are never actually put in the definition of f_x and the reader is left to "derive the new expression" themselves. This also introduces so much unnecessary extra text to the article, where math would do the trick (quite literally) in only a single line. Perhaps the authors want to rethink their approach around how f_x is incorporated in 2.3 and reduce the overall amount of text significantly in favor of clearer math.

Reply: We found this suggestion beneficial.
We have added further equations to section 2.3 which explicitly show the resulting modifications included in the distribution function and allowed us to shorten the verbal explanations as requested. For the sake of clarity, we enclose here the related paragraph (page 10):

> "We assume that the openness-to-change vs. conservation dimension (i) is relevant for agents' tendency to deviate from social norms and their need for autonomy and (ii) affects employees' shirking behaviour ($s_{i,t}$). The self-transcendence/self-enhancement dimension determines cooperativeness and responsiveness to rewards, influencing cooperative decisions ($c_{i,t}$) in our model.
> The amount of time spent on shirking is derived from a triangular distribution with parameters as defined in equations 6-8.
>
> $$a_{i,t} = s_t^*(1 - \delta_i) \quad (6)$$
> $$b_{i,t} = s_t^*(1 + \delta_i) \quad (7)$$
> $$m_{i,t} = s_t^* + \phi_i \quad (8)$$
>
> Analogously, the triangular distribution parameters determining time spent on cooperation is as follows:
>
> $$a_{i,t} = c_t^*(1 - \delta_i) \quad (9)$$
> $$b_{i,t} = c_t^*(1 + \delta_i) \quad (10)$$
> $$m_{i,t} = c_t^* + \gamma_i + \rho_i \quad (11)$$
>
> The actual time an employee spends on individual tasks is defined residually after $s_{i,t}$ and $c_{i,t}$ are randomly drawn from the triangular distribution:
>
> $$p_{i,t} = \tau - s_{i,t} - c_{i,t} \quad (12)$$
>
> The following subsections describe how we model these assumptions and how they relate to agents' behaviour."

Reviewer Point P 1.10 — What's the reason to choose 500 days as the total simulation time? Was there any criterion for convergence employed? Do the model dynamics stabilize?

Reply: Thank you very much for raising this issue. Please refer to our answer to comment P 2.2.

Reviewer Point P 1.11 — Sect. 4 is very good. Short, to the point, and highlight all novelties excellently.

Reply: Thank you very much for these kind words.

Technical comments

Reviewer Point P 1.12 — References are printed with bracket notation, e.g. [7]. However, starting a sentence with such a reference is not appropriate style (e.g., "[7] call for the development...") in second paragraph). The authors should use last name and et al. followed by the refernce. E.g., Chatman et al. [7] call for the development....

Reply: We thank you very much for this advice.
The references have been modified according to your suggestion.

Reviewer Point P 1.13 — It is easier to the reader if the same word is used for the same notion consistently throughout the text. It seems that e.g., "company" and "firm" are used interchangeably. Eliminating different words used for the same concept will make the text easier to read and follow.

Reply: Following the referee's advice, the word "firm" has now been replaced with "company" throughout the whole paper.

Reviewer Point P 1.14 — Equations (4,5) etc. started to become difficult to follow because of the way the time indexing works. At the start of Sect. 2.1 the authors say that "for notational convenience they do not use a time index", which is a decision I certainly do not agree with. Then, in (4) a secondary subscript (-1) is used for the time index of the previous step, even though the variables of the current time step have no time index. Furthermore, the sudden appearance of an asterisk * for the t variables makes things even more confusing. It is not explained in text what the asterisk represents until it is too late. Only when one reaches equation 7 it becomes clear that the asterisks denote some mean value. I think the mathematical exposition of the formulas can be improved for more clarity. I also wonder, is it indeed better to have t with subscripts for all task times, instead of having three variables, p c and s instead?

Reply: Yes, you are absolutely right. The absence of a time index might be a source of inconsistency. For this reason, we decided to modify the notation of the variables you mention throughout the manuscript by including a time subscript t as follows:

1. $t_p \Rightarrow p_t$
2. $t_c \Rightarrow c_t$
3. $t_s \Rightarrow s_t$
4. $t_p^* \Rightarrow p_t^*$
5. $t_c^* \Rightarrow c_t^*$
6. $t_s^* \Rightarrow s_t^*$

However, the use of the asterisk $*$ is a deliberate choice as it is intended to denote the social norm to which agents' behaviour is anchored. The referee's observation is correct because depending on the number of peers each agent has, the social norm can be identical with the mean of time spent on each activity across all agents. In our core simulation we assume that social norms are the same for all agents (i.e. a "Global" norms environment), resulting in the number of agent's peers to be equal to the number of agents in the model. Hence in this case for any social norm $n_t^* = \bar{n}_t$ holds true. This is not the case in simulations with a lower degree of connectivity

among the agents (i.e. "Neighbours" and "Random" norms environments, see Supporting Information S2), where an agent's observed norms are influenced only by a subset of all agents. To make this crucial distinction clearer, we have added a few clarifying sentences at page 7:

> "For the sake of generalisation, we use an asterisk to denote social norms independently of their scope. Since social norms coincide with the average time spent on any activity across the whole population in a *Global* scope of social norms, it might be possible to adopt the usual notation for averages (i.e. $\bar{c}_t$ or $\bar{s}_t$). However, this would not be valid for the two additionally tested local scopes (see Supporting Information S2). Hence our use of the asterisk which avoids this potential source of notational inconsistency."

Reviewer Point P 1.15 — Why is f_x written f_x instead of $f(x)$, given that it is a function of x?

Reply: Thank you for pointing this out.
As already stated in P 1.7, we have decided to delete equation (i) defining the generic triangular distribution.

Reviewer Point P 1.16 — After Fig. 1: "hence the mode is equal to the social norm" please say the "mode of the distribution".

Reply: Yes, we did this, see page 8.

Reviewer Point P 1.17 — "Factor analyses show that the basic values can be aggregated along two dimensions." Needs a citation.

Reply: Yes, you are absolutely right. We have added the citation appropriately.

Reviewer Point P 1.18 — "deviations from the shirking norm and the cooperation norm" I believe throughout the text such references can be improved by explicitly referencing the associated symbol. If I have understood the methodology, the cooperation norm is just t_c^*. Hence, this symbol should be added immediately after "cooperation norm".

Reply: Thank you for this comment. We added the symbols associated to the shirking (s_t^*) and cooperative (c_t^*) norms throughout the manuscript as requested. We hope you find the text better referenced.

Reviewer 2

Reviewer Point P 2.1 — The choice of the four employee types (ST, SE, C, and O) needs further justification. The types are on two independent dimensions (ST-SE, and C-O). Thus, an employee can be open to change/conservative AND self-transendent/self-enhancing. Therefore, the more intuitive types would be ST-O, ST-C, SE-O, and SE-C. This would change values in Table 1 and density functions, potentially simplifying the model because there will be no intermediate distribution curves anymore.

Reply: We thank the referee for raising these concerns.
We perfectly agree with the existence of mixed types. In fact, the Schwartz theory has 12 individual values which are all mixes of the two dimensions. Having mixed value types might allow us to have agents reacting to all the attributes discussed in Section 2.3 and to avoid intermediate distribution curves, instead of assuming that C/O types are indifferent to rewards and that SE/ST ones do not react to monitoring strategies. However, choosing the four *pure* types is a strategic simplification and is more in line with the concept of *value hierarchy* advocated by the Schwartz theory of values. Adopting this strategy also allowed us to *isolate* the effects of each value dimension with respect to each attribute of interest. By doing so, we had been able to identify O-agents as the main drivers of behaviour, as discussed in Section 3. Assuming mixed types as you suggest, instead, might have required a further and unnecessary step of analysis to disclose such an important result, *ex-post*, or it even might have been missed at a first glance, *ex-ante*.
Moreover, mixed types emerge at an aggregate level when looking at the shares of the population. We firmly believe that aggregating the value dimensions leads to (i) more robust results since individual errors cancel out and (ii) to a less challenging future empirical validation.

However, since this issue could have been not clearly stated, we added the following sentence in Section 2.3 at page 10:

> "To have a clearer understanding of the dynamics of the model, we do not assume the existence of mixed types arising from the combination of the two aforementioned dimensions (ST-O, ST-C, SE-O, SE-C). For the purpose of this paper, we find it convenient to isolate the impact of each value dimension on each attribute."

We sincerely hope this answer is acceptable to you.

Reviewer Point P 2.2 — Additionally, the choice for the length of simulations (500 days) is not discussed. Plots show that populations do not reach equilibrium until the end of the simulations (although they do not seem to be changing course either).

Reply: Thank you very much for raising this important issue. While our main focus of investigation lies in the emerging dynamics of the different scenarios, it is arguably the case that the stopping point of the simulations (after 500 steps/working days) is somewhat arbitrarily chosen. To provide a more complete picture of the convergence of scenarios as well as their resulting long-run steady states, we have now extended all our simulations to run for 3000 steps instead. From the results of these prolonged simulation runs, it should now become clear to the reader that (i) the variable levels stabilise and (ii) no further dynamics emerge even after such a long time.

Because of the fundamental nature of this change, we had to incorporate a multitude of changes in various places of our work. Most notable changes can be found in the Results section (Section 3) and the appendix discussing the sensitivity analysis (Supporting Information S3). Please note that while our core findings remain the same, we also had to add a few paragraphs on the long-run steady states of the different scenarios. While we did our best to keep those as concise as possible, they still lengthened the paper a bit.

Reviewer Point P 2.3 — O-agents are important drivers of the evolution of social norms because of their larger variance in deviating from norm compared to other employees. Figure 9 proves this effect. At first, it sounds counter intuitive that the positive and negative deviations from the norm do not cancel each other out. Since this is such an important determinant of the model behavior, it deserves a more detailed explanation than the single sentence given on page 20.

Reply: We would like to bring to the referee's attention the following paragraph that already appears in the manuscript in Section 3.1 (page 19):

> "It is not immediately obvious why C-agents produce more than O-agents, although both spend the same time on their individual tasks on average. The reason is that the variation in O-agents' behaviour is larger than the variation in the behaviour of C-agents. In every period, some O-agents choose an unfavourable combination of high shirking and high cooperation, which leads to low individual task time. Although this is compensated by others, who choose low shirking and low cooperation, the effect is not symmetric due to the decreasing marginal product of individual task time. Higher variation hence leads to lower group output despite the roughly equal average behaviour of the two groups. We checked this hypothesis by lowering the variation in O-agents' behaviour. If the variation gets closer to the one of C-agents, the O-agents' realised output approaches the one of C-agents."

We believe that this paragraph already provides sufficient explanations for the larger O-types' variance driving our counter intuitive result. Moreover, we prefer not to add additional sentences, because *Reviewer 1* has asked us to shorten the text. But if you think that this is not sufficient, we are willing to make some modifications.

Reviewer Point P 2.4 — Figure quality is too poor and I had difficulty reading the labels. Using a vector format for saving images (e.g. PDF) should solve this.

Reply: Thank you for pointing this out.
We share your concern about the readability of the plots, which originally had been produced in PDF. The reason why we have not submitted the plots in PDF lies on the figure requirements imposed by the journal. Downloading each plot separately greatly enhances the quality of the provided figures. We assume that this reduction in figure quality was made to reduce the size of the manuscript's PDF and that the final publication will provide higher quality figures.

Reviewer Point P 2.5 — The Discussion benefits from expanding on the limitations of the study. For example, although the study incorporates heterogenous agents instead of a single type, there are other parameters kept fixed that could affect the results. Tasks are not homogenous either, some requiring more collaboration and some less. What would be the effect of a mixed management style depending on the amount of collaboration needed for a task? I suppose in such scenarios, TrustComp would be a winning strategy.

Reply: Thank you very much for this interesting comment.

We might provide two potential answers. First, if you refer to having different *degree of task interdependence* (κ), we have provided some tests in the sensitivity analysis. Indeed, at page 9 of Supporting Information S3 we claim the existence of a negative correlation between a higher κ and *Competitive* and *Trusting* scenarios, since the higher partial contribution of cooperative activities to the Cobb-Douglas output function. As a consequence, the combined *Trustcomp* scenario can not be categorised as a *winning strategy* since it shows even stronger decreasing dynamics of output.
Second, if you refer to having heterogeneous κ at an individual or group level, please refer to comment P 1.4.
We agree that changing these assumptions would have an impact on the results. However, since the paper is already quite long we refrain from a more detailed version of the manuscript.

In any case, we completely agree about expanding Section 4 with the inclusion of some limitations of the model. For this reason, we added the following sentence at page 26, which we hope you find appropriate.

> "Further attempts to overcome some limitations of this model might include (i) endogenous management strategies, (ii) heterogeneous tasks, skills and productivity levels and (iii) adaptive agents' behaviour in a more complex network environment. This is left for future research. "
