# Supplementary material for: A value-based model of job performance
Source: PLoS One. 2022 Jan 27;17(1):e0262430. doi: 10.1371/journal.pone.0262430 (PMC8794576; doi:10.1371/journal.pone.0262430)
Supplement: Attachment — Submitted filename: Response to Reviewers.pdf [file pone.0262430.s003.pdf]

Response to Reviewers

"A value-based model of job performance"

(PONE-D-21-29609)

We would like to sincerely thank the referees for their comments and suggestions and to have deemed our first-revision responses as satisfactory.
We address the Reviewer 1's minor comments in the following pages.

Reviewer 1

Minor Comments

Reviewer Point P 1.1 — In equation 2 the subscript of $\bar{c}$ on the left-hand-side of the equation should be i, t instead of j, t. And this is also true for equation 1. Given that equation 2 defines $\bar{c}$ so that the right-hand-side is NOT a function of j, as j is reduced via summation, it cannot be that the left-hand-side is a function of j either.

Reply: Thank you very much for raising this concern. We have now changed equations 1 and 2 according to your advice as follows.

> The output of employee i is given by a function of the Cobb-Douglas type and depends on the time devoted to the employee's own work, $p_{i,t}$, and the average time other team members devote to cooperation, $\bar{c}_{i,t}$, defined in equation 2.
>
> $$O_{i,t} = p_{i,t}^{1-\kappa} * \bar{c}_{i,t}^{\kappa} \quad (1)$$
>
> [...]
>
> $$\bar{c}_{i,t} = \frac{1}{(N-1)} \sum_{j \neq i} c_{j,t} \quad (2)$$

Reviewer Point P 1.2 — Equation (4) must end with a period "."

Reply: Thank you for pointing out this punctuation inconsistency. However, we believe that equation 4 does not need a full stop as the preceding sentence already contains one. Since this could be unclear, we have arranged that sentence differently and it now looks as follows.

There is also a norm for helping others and participating in group activities, c_t^*.

$$c_t^* = (1 - h)\ c_{t-1}^* + h\ \frac{\sum_{j \epsilon N} c_{j,t-1}}{N} \tag{4}$$

Reviewer Point P 1.3 — Page 7 first paragraph ends with: ", and the average of all agents' behaviour in the previous period:" So it ends with colon, but nothing follows the colon; no equation but rather a new paragraph.

Reply: Thank you for pointing this out. We have now fixed the punctuation of the paragraph you mention.

Reviewer Point P 1.4 — Equation (5) must end with a period "."

Reply: We have changed the preceding sentence such that no full stop is required in equation 5.

> From these norms, the social norm for individual working time follows from the time constraint, see equation 5.
>
> $$p_t^* = \tau - s_t^* - c_t^* \tag{5}$$

The same has been done for the other equations in the model, for the sake of consistency.

Reviewer Point P 1.5 — When introducing the triangular distribution $x_t \sim T(a, b, m)$ please say that m is the mean. Furthermore, please write "the natural bound $a = m - m = 0$" (i.e., explicitly add " $= 0$")

Reply: Yes, you are absolutely right. We have clarified in the text that m stands for the *mode* of the triangular distribution and explicitly added the equality to zero of the lower bound a.

> The actual time employees spend on a generic activity at a given time x_t is derived as a random draw from the triangular distribution, that is $x_t \sim T(a, b, m)$ where m is the mode of the distribution, see Figure 2. [...] The natural lower bound is $a = m - m = 0$ because employees cannot allocate negative amounts of time to an activity.
